# Warming increases survival and asexual fitness in a facultatively sexual freshwater cnidarian with winter diapause

**DOI:** 10.1002/ece3.9981

**Published:** 2023-04-10

**Authors:** Jácint Tökölyi

**Affiliations:** ^1^ MTA‐DE “Momentum” Ecology, Evolution & Developmental Biology Research Group, Department of Evolutionary Zoology University of Debrecen Debrecen Hungary

**Keywords:** climate change, Cnidaria, freshwater ecology, life history, population dynamics, seasonality

## Abstract

Temperature is a key abiotic factor controlling population dynamics. In facultatively sexual animals inhabiting the temperate zone, temperature can regulate the switch between asexual and sexual modes of reproduction, initiates growth or dormancy, and acts together with photoperiod to mediate seasonal physiological transitions. Increasing temperature due to recent global warming is likely to disrupt population dynamics of facultatively sexual animals because of the strong temperature dependence of multiple fitness components. However, the fitness consequences of warming in these animals are still poorly understood. This is unfortunate since facultatively sexual animals—through their ability for asexual reproduction resulting in quick population growth and sexual reproduction enabling long‐term persistence—are key components of freshwater ecosystems. Here, I studied the fitness effects of warming in *Hydra oligactis*, a freshwater cnidarian that reproduces asexually throughout most of the year but switches to sexual reproduction under decreasing temperatures. I exposed hydra polyps to a simulated short summer heatwave or long‐term elevated winter temperature. Since sexual development in this species is dependent on low temperature, I predicted reduced sexual investment (gonad production) and elevated asexual fitness (budding) in polyps exposed to higher temperatures. The results show a complex effect of warming on sexual fitness: While gonad number decreased in response to warming, both male and female polyps exposed to high winter temperature were capable of multiple rounds of gamete production. Asexual reproduction and survival rate, on the contrary, clearly increased in response to higher temperature, especially in males. These results predict increased population growth of *H. oligactis* in temperate freshwater habitats, which will likely affect the population dynamics of its main prey (freshwater zooplankton), and through that, the whole aquatic ecosystem.

## Graphical abstract

*Hydra oligactis* polyps were exposed to simulated summer heatwaves and/or elevated winter temperature. High temperatures had complex (both negative and positive) effects on components of sexual reproduction, while overall survival rate and asexual fitness increased. These results predict increased population growth of hydra polyps in a warming world.

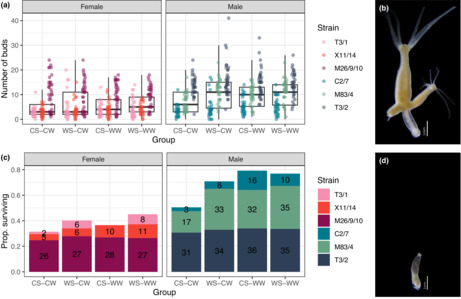

## 1 INTRODUCTION

Organisms living in the temperate zone are exposed to seasonal fluctuations in temperature that provide contrasting physiological environments during summer and winter. Many species inhabiting such environments have adapted to temperature variation by specializing and evolving thermal preference to either high or low temperature (Huey & Kingsolver, 1989) or scheduling their major life events to different parts of the year (McNamara & Houston, 2008). For instance, among invertebrates that combine sexual with asexual reproduction in a complex life cycle (i.e., facultatively sexual organisms, such as aphids, flatworms, crustaceans, rotifers, sponges, or cnidarians), sexual and asexual reproduction events are often performed in different parts of the year (Acker & Muscat, 1976; Fuchs et al., 2014; Green, 1966; Reisa, 1973; Shaffer et al., 2020; Simon et al., 2002). Increasing or decreasing temperatures in these species can induce a switch from asexual to sexual reproduction (Acker & Muscat, 1976; Fuchs et al., 2014; Reisa, 1973; Schröder, 2005; Shaffer et al., 2020; Simon et al., 2002; Vowinckel, 1970), initiate the growth and development of embryos inside resting eggs (Cáceres & Schwalbach, 2001; Cooley, 1971; Gilbert, 2017; Gulbrandsen & Johnsen, 1990; Hairston & Kearns, 1995; Vandekerkhove et al., 2005), and act as a modulator of other cues, such as photoperiod and crowding (Decaestecker et al., 2009; Gyllström & Hansson, 2004; Innes, 1997; Reisa, 1973; Schröder, 2005).

Temperature is a key regulator of the dynamics of populations because it determines locomotor activity and food intake rates (Angilletta et al., 2002) and acts as a physical factor regulating cellular processes, such as metabolism or energy budgets (Brown et al., 2004; Huey & Kingsolver, 2019), ultimately determining growth, reproduction, and survival (Angilletta, 2009). In facultatively sexual organisms, both sexual and asexual modes of reproduction critically depend on temperature and switches in the mode of reproduction are expected to have a major influence of population dynamics. Both modes of reproduction contribute to population persistence and/or growth, albeit in different ways. Asexual reproduction results in quick population growth, allowing genotypes to increase in frequency when conditions are favorable (Scheuerl & Stelzer, 2019). Sexual reproduction, by contrast, often results in the production of resting eggs that are able to survive adverse conditions. These resting eggs can persist for years or even decades and replenish populations after stochastic extinction events (Franch‐Gras et al., 2017), provide sources of novel genetic combinations with higher fitness (McLean et al., 2022), and enable dispersal of propagules to novel habitats (Panov et al., 2004).

Recent climate change, however, is affecting temperatures worldwide, and rapidly rising temperatures have negative effects on organisms that evolved under a different thermal regime (McCarty, 2001; Walther et al., 2002). Documented effects of global warming include changes in phenology that is, timing of major life history events such as migration in birds or flowering in plants (Jenni & Kéry, 2003; Molnár et al., 2012), shifts in geographical range size (Thomas, 2010), and a widely detected decline in animal body size (Sheridan & Bickford, 2011). Facultatively sexual organisms, due to their adaptation to seasonally varying temperature regimes, should be strongly impacted by warming temperatures, although the complexity of facultatively sexual life cycles makes prediction of the expected consequences of global warming difficult and little is known about the expected consequences of climate warming in these organisms. In aphids, for instance, documented population responses to warming can be either positive, neutral, or negative (Blanchard et al., 2019; van Baaren et al., 2010). Since aphids are reproducing parthenogenetically during summer and switch to sexual reproduction at the approach of the winter, rising temperatures are expected to yield short‐term increases in population sizes (until temperature becomes stressful) and the disappearance of sexually produced offspring (Blanchard et al., 2019). Warming temperatures have been shown in mesocosm experiments to boost spring population growth of zooplankton, with a higher effect in parthenogenetically reproducing cladocerans than nonparthenogenetic copepods (Ekvall & Hansson, 2012). Due to the altered population growth and differential thermal sensitivity, zooplankton communities not only experience changes in dominance patterns (Ekvall & Hansson, 2012) but also experience trophic mismatches that can result in population declines (Winder & Schindler, 2004). However, despite these examples, the number of studies examining the expected consequence of temperature warming on facultatively sexual organisms is still very low. This is unfortunate because facultatively sexual organisms are key components of the ecosystems they inhabit due to their ability to achieve quick population growth through asexually reproduction and long‐term persistence and dispersal through the production of dormant stages. Therefore, changes in their population dynamics due to climate warming are likely to have wide‐range consequences at the level of the whole ecosystem.

**FIGURE 1:**
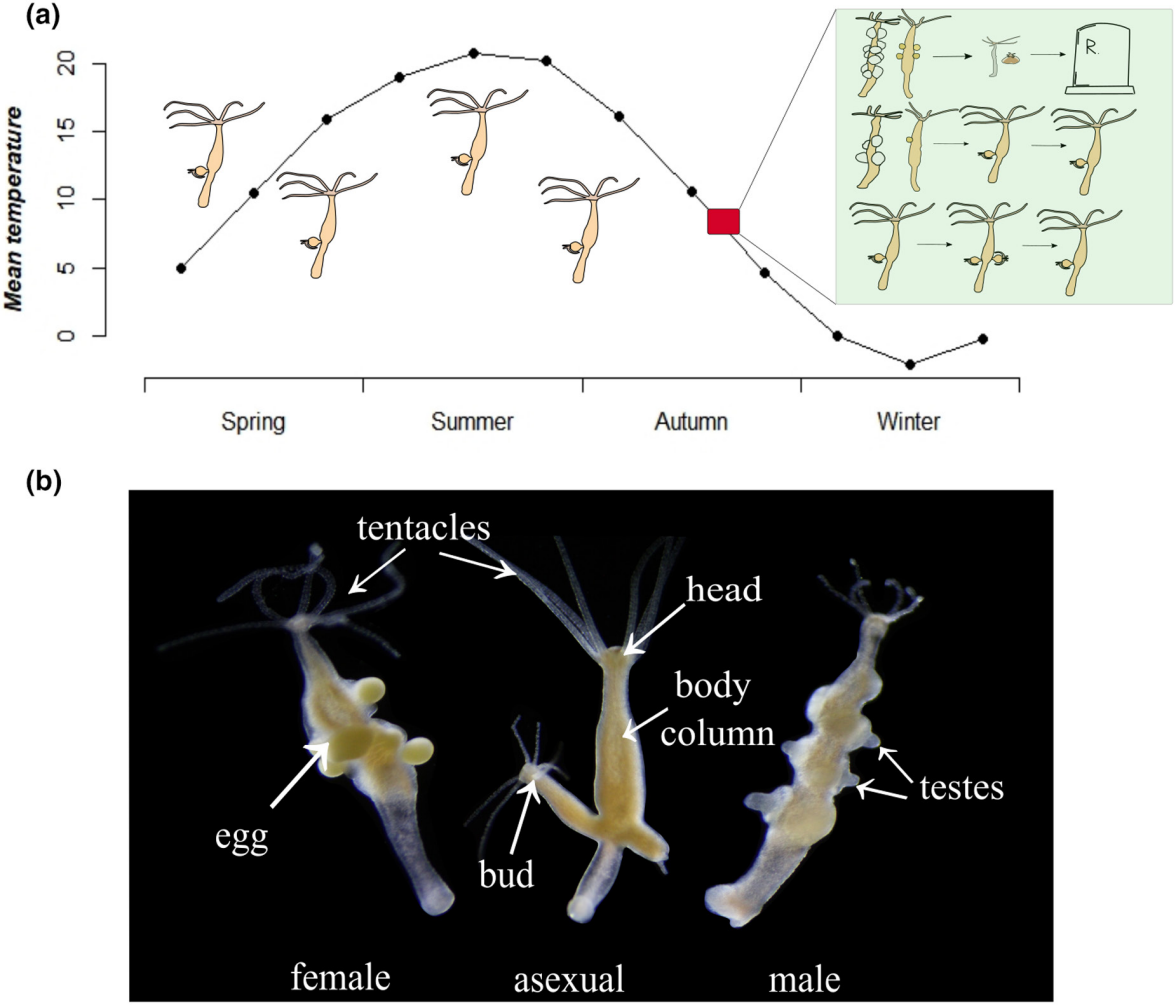
(a) Throughout most of the year, *Hydra oligactis* polyps reproduce asexually. Before the onset of winter, some polyps switch to a sexual mode of reproduction. Sexual individuals undergo senescence and some of them die, while others regenerate and revert to asexual reproduction. (b) Photographs of asexual and sexual polyps. Image and graphics credit: Erzsébet Ágnes Nehéz.

The genus Hydra is a group of sessile, predatory freshwater cnidarian species that inhabit all continents except Antarctica (Schuchert, 2010). They are considered important freshwater bioindicator species and frequently used in ecotoxicological research (Cera et al., 2020). Hydra species differ in thermal sensitivity, with some of them (e.g., *H. vulgaris, H. circumcincta*, and *H. viridissima*) tolerating high temperatures that occur in tropical environments or shallow temperate freshwater bodies during the summer (Reisa, 1973). Other species, such as *H. oligactis* and *H. oxycnida*, on the contrary, are cold‐adapted species that prefer low temperatures, and even short‐term exposures to temperatures above 30°C are lethal (Bosch et al., 1988). The species used in this study, *Hydra oligactis*, reproduces asexually throughout much of the year through budding, but switches to a sexual mode of reproduction upon exposure to cold temperatures (Figure 1a; Reisa, 1973). Males produce testes, and females produce ovaries (Figure 1b), and upon fertilization the egg develops into a resting embryo surrounded by a thick shell that is extremely resistant to adverse conditions (Reisa, 1973). Hence, the production of resting eggs appears to be an adaptation to survive freezing water through dormancy, and indeed, sexually reproducing individuals are found in nature during autumn, before the freezing of water surface occurs (Miklós et al., 2021; Sebestyén et al., 2018). Following sexual reproduction, hydra polyps experience a senescence‐like process and increased mortality risk, although some of them survive and revert to asexual reproduction (Tökölyi et al., 2017; Yoshida et al., 2006), depending on their age, size, and genotype (Miklós et al., 2022; Ngo et al., 2021; Sebestyén et al., 2020). Because of the presence of these asexual individuals, *H. oligactis* can reach huge population densities during late winter and early summer (Bryden, 1952; J.T. personal observation). This, in turn, could have major consequences for the population dynamics of their prey (mainly cladocerans and copepods, but also small fish; Cuker & Mosley, 1981; Deserti et al., 2017; Elliot et al., 1997; Massaro et al., 2013; Rivera‐de la Parra et al., 2016; Schwartz et al., 1983) in a manner that amplifies with climate change. However, whether warming temperatures affect sexual or asexual fitness, and thereby population dynamics in hydra is still unclear.

Here, I set out to address this question by exposing *H. oligactis* polyps belonging to six strains (three male and three female) to elevated temperature. I hypothesized that warming would affect physiological processes involved in reproduction and survival in hydra, with detectable effects on fitness components (sexual reproduction, asexual reproduction, and survival). Since climate warming is predicted to be unequal across seasons (Meehl et al., 2007), I considered two scenarios: an increased frequency of summer heatwaves and an increased average winter temperature, both of these predicted based on current climate projections for the temperate and boreal zone (Meehl et al., 2007). To this end, hydra polyps were exposed either to a short (1‐week long) period of high summer temperature (23°C, compared to 18°C for controls), which was followed by a winter treatment consisting either of low (8°C) or high winter temperature (12°C) maintained for 5 months. Throughout the experiment, sexual and asexual fitness components were recorded. Because of the sensitivity of *H. oligactis* to high temperature, I predicted reduced performance (both sexual and asexual) of polyps exposed to a short heatwave. Furthermore, because of the dependence of sexual reproduction on cold temperatures in *H. oligactis*, I predicted a reduced sexual fitness in groups exposed to warm winter temperatures. Conversely, a higher asexual fitness and survival might be predicted due to the negative relationship between sexual reproduction and other fitness components (Ngo et al., 2021; Roff, 1993; Stearns, 1989).

## 2 MATERIALS AND METHODS

### 2.1 Strains and culture conditions

For the experiment presented here, six *H. oligactis* strains were used (three male strains: C2/7, M83/4, and T3/2 and three female strains: X11/14, M26/9/10, and T3/1). All strains were established from a single individual that was collected from freshwater bodies in Eastern Hungary between 2016 and 2020 and asexually propagated in the laboratory under simulated summer conditions (Gergely & Tökölyi, 2023). These conditions include 18°C temperature and 12/12‐h light/dark cycle. Polyps were kept individually in 6‐well tissue culture plates containing artificial hydra medium (1 mM Tris, 1 mM NaCl, 1 mM CaCl_2_, 0.1 mM KCl, 0.1 mM MgSO_4_; pH: 7.6), and they were fed and cleaned four times per week.

### 2.2 Experimental design

All data recorded for this experiment come from polyps that were kept individually. The experiment consisted of two treatments: a summer treatment and a winter treatment. The summer treatment lasted for 1 week and aimed at simulating a heatwave. The winter treatment lasted for 5 months and aimed at simulating elevated winter temperature. For both treatments, the control values were chosen such that both summer and winter conditions are comparable to our previous studies investigating *H. oligactis* life history. Hence, the control temperature for summer was set as 18°C (control summer, CS) and the control temperature for winter was set to 8°C (control winter, CW). To simulate a summer heatwave, I exposed polyps to 23°C for 1 week (warm summer, WS). Water temperatures as high as 25°C regularly occur in natural populations at the end of the summer (J. T. personal observation), however, in the laboratory larger or longer heat treatments (i.e., above 23°C or longer than one week) result in excess mortality. For the winter treatment, I exposed experimental polyps to 12°C temperature for 5 months (warm winter, WW). A four degree increase in winter temperature is in accordance with IPCC predictions for Europe by the end of the century (Meehl et al., 2007). The summer and winter treatments were applied in a factorial design, such that there were four experimental groups: control summer–control winter (CS‐CW); control summer–warm winter (CS‐WW); warm summer–control winter (WS‐CW) and warm summer–warm winter (WS‐WW).

During the 1‐week summer treatment (either 18 or 23°C), polyps were fed four times per week. After the summer treatment, polyps were photographed on a 1‐mm grid paper sheet to measure their size, since size at cooling is an important predictor of sexual reproduction and postreproductive survival (Ngo et al., 2021). Next, they were moved to either 8 or 12°C, depending on their treatment and an 8/16‐h light/dark cycle. Lowering temperature induces sexual reproduction in this species, and I recorded the presence of male or female gonads four times per week as a proxy for sexual fitness. In addition, the number of detached buds was recorded twice per week as a proxy for asexual fitness. The winter treatment lasted for 5 months during which polyps were fed and cleaned twice per week. A 5‐month winter treatment is necessary and sufficient time period to quantify sexual reproduction and postreproductive survival in most polyps in these strains. At the end of the winter treatment, polyps were scored as survived if they had an intact body with tentacles and had the ability to feed, and they were scored dead if they disappeared during the experiment or consisted only of necrotic tissue. All experimental groups were started with 30–36 polyps per strain, with a total starting sample size of *N* = 843, although some individuals were excluded because they were accidentally lost during the experiment or they proved to be sex‐changed individuals (see Results). The experiment was initiated in batches over a period of about 2 months, depending on the availability of experimental animals. Each batch consisted of animals randomly divided into four treatment groups, and any batch effects were statistically taken into account (see below).

### 2.3 Data analysis

The effects of treatments were analyzed for the following dependent variables: (1) body size after summer treatment; (2) sexual development time (days elapsed after the beginning of the winter treatment until the appearance of first gonads); (3) sexual fitness (total number of eggs produced by females and the maximum number of testes seen on a male); (4) asexual fitness (total number of buds produced by an individual from the start to the end of the experiment, that is, during both the summer and winter treatments); and (5) survival rate. I used generalized linear mixed models implemented in the glmmTMB package (v1.1.4) in R version 4.2.2 (Brooks et al., 2017; R Core Team, 2020). Gaussian errors were assumed for body size and binomial errors for survival rate. For sexual development time, sexual and asexual fitness (count data), four different error structures were considered: Gaussian, Poisson, negative binomial with linear parametrization (*nbinom1*), and negative binomial with quadratic parametrization (*nbinom2*; Brooks et al., 2017). The model best fitting to the data was selected with Akaike Information Criteria corrected for small sample size (AICc). After selecting the right distribution, I fitted a model with experimental treatment as a fixed effect and strain ID and batch ID as random effects. This model was compared with a random effects null model via likelihood ratio tests (LRTs). *Post hoc* treatments‐versus‐control comparisons with Dunnett's correction were obtained using the *emmeans* v1.8.3 R package (Lenth, 2022). For the model analyzing body size after summer treatment, only one predictor was included (summer treatment) and I used LRT to evaluate the effect of treatment on size. Finally, I generated model diagnostics plot using the DHARMa v0.4.5 R package (Hartig, 2022) to check that model residuals did not show outstanding deviations from model assumptions. Since the model diagnostics for sexual development time showed substantial deviations from the expectation for both males and females, I also analyzed these variables with nonparametric Kruskal–Wallis tests, followed by pairwise Wilcoxon tests with Holm's correction.

## 3 RESULTS

Of the total 843 individuals, 12 were accidentally lost during routine handling and nine individuals were sex‐changed. All data were removed for these individuals, yielding a sample size of 822, with 206 individuals in the CS‐CW group, 200 individuals in the WS‐CW group, 210 individuals in the CS‐WW group, and 206 individuals in the WS‐WW group.

### 3.1 Body size after summer treatment

**FIGURE 2:**
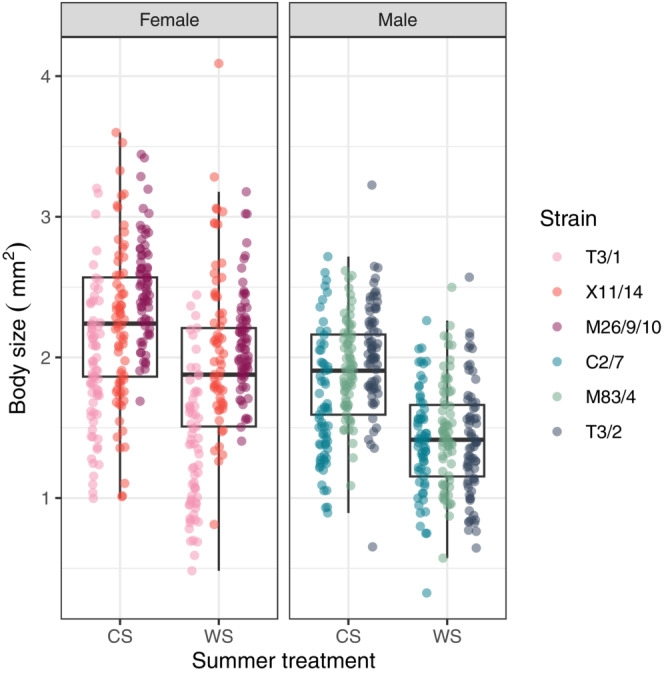
Body size of *Hydra oligactis* polyps from three male and three female strains and cultured for one week on 18°C (control summer, CS) or exposed to a simulated one‐week heatwave of 23°C (warm summer, WS). A simulated summer heatwave significantly reduces both male and female body size (see Results).

A one‐week heat treatment reduced body size in male polyps by approximately 17% and female polyps by approximately 24%, with the difference being statistically highly significant in both sexes (Gaussian GLMM, LRT males: chi‐square = 117.59, *p* < .001; females: chi‐square = 42.984, *p* < .001; Figure 2).

### 3.2 Sexual development time

**FIGURE 3:**
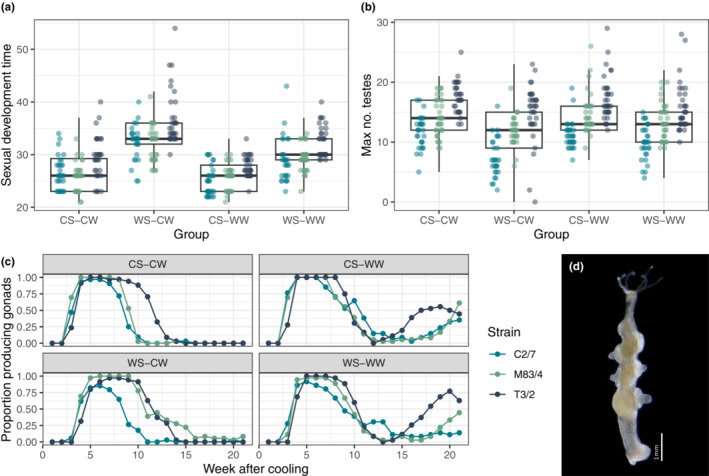
Sexual reproduction in male *Hydra oligactis* polyps belonging to three distinct strains exposed to four distinct temperature regimes: CS‐CW (18°C summer–8°C winter; control group), WS‐CW (23°C summer–8°C winter), CS‐WW (8°C summer–12°C winter) or WS‐WW (23°C summer–12°C winter). The summer treatment lasted for one week, while the winter treatment lasted 21 weeks. The graph shows the time elapsed until the appearance of the first gonads after cooling (a), the maximum number of testes (b) and temporal changes in the frequency of sexual individuals (c). A photograph of a male individual with mature testes is shown on panel d for illustration.

**FIGURE 4:**
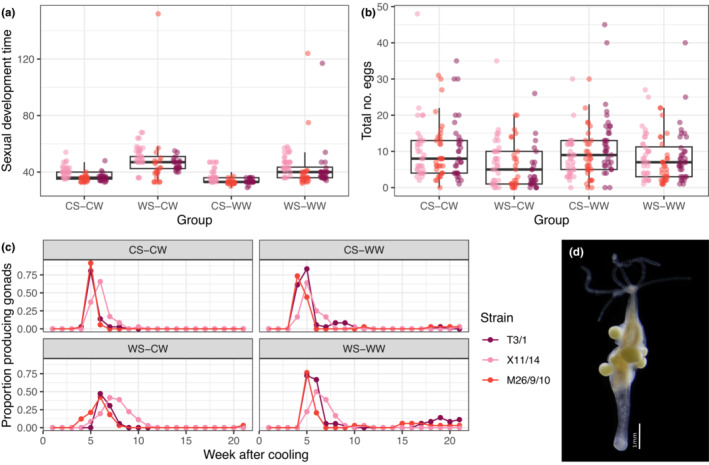
Sexual reproduction in female *Hydra oligactis* polyps belonging to three distinct strains exposed to four distinct temperature regimes: CS‐CW (18°C summer–8°C winter; control group), WS‐CW (23°C summer–8°C winter), CS‐WW (8°C summer–12°C winter) or WS‐WW (23°C summer–12°C winter). The summer treatment lasted for one week, while the winter treatment lasted 21 weeks. The graph shows the time elapsed until the appearance of the first gonads after cooling (a), the total number of eggs (b) and temporal changes in the frequency of sexual individuals (c). A photograph of a female individual with mature eggs is shown on panel d for illustration.

In both male and female strains, sexual development time was significantly influenced by treatment (males: LRT, Gaussian GLMM, *λ* ^2^ = 257.19, *p* < .001; females: LRT, negative binomial GLMM with quadratic parametrization, *λ* ^2^ = 133.34, *p* < .001). Both male and female polyps exposed to 1‐week heat treatment showed delayed sexual development, while those exposed to elevated winter temperatures showed slightly, but significantly faster sexual development (Table 1, Figures 3 and 4). Polyps in the WS‐CW group delayed their reproduction most (Figures 3a and 4a), implying that a large sudden drop in temperature has negative effects on sexual development. The results were corroborated by the Kruskal–Wallis test and the pairwise Wilcoxon test as *post hoc* analysis, with the exception of males in the CS‐WW group, where advancement of sexual development time was only marginally significant (*p* = .081).

**TABLE 1:** Sexual development time, sexual fitness (number of gonads), asexual fitness (number of buds) and survival rate of male and female *Hydra oligactis* polyps exposed to summer heatwaves (WS‐CW), elevated winter temperature (CS‐WW), or both (WS‐WW), compared with polyps exposed to a control summer–control winter scenario (CS‐CW).

Contrast	Males			Females		
	Estimate	SE	*p*‐value	Estimate	SE	*p*‐value
Sexual development time	*gaussian*			*nbinom2*		
(WS‐CW)–(CS‐CW)	**6.53**	**0.44**	**<.001**	**0.26**	**0.03**	**<.001**
(CS‐WW)–(CS‐CW)	**−1.04**	**0.43**	**.047**	**−0.08**	**0.03**	**.016**
(WS‐WW)–(CS‐CW)	**3.01**	**0.44**	**<.001**	**0.13**	**0.03**	**<.001**
Sexual fitness	*gaussian*			*nbinom1*		
(WS‐CW)–(CS‐CW)	**−2.89**	**0.43**	**<.001**	**−0.61**	**0.12**	**<.001**
(CS‐WW)–(CS‐CW)	−0.03	0.43	.998	−0.04	0.10	.910
(WS‐WW)–(CS‐CW)	**−1.32**	**0.43**	**.007**	−0.21	0.10	.082
Asexual fitness	*nbinom1*			*nbinom2*		
(WS‐CW)–(CS‐CW)	**0.30**	**0.07**	**<.001**	0.19	0.10	.114
(CS‐WW)–(CS‐CW)	**0.24**	**0.07**	**.001**	0.13	0.09	.387
(WS‐WW)–(CS‐CW)	**0.28**	**0.07**	**<.001**	0.21	0.09	.067
Survival	*binomial*			*binomial*		
(WS‐CW)–(CS‐CW)	**1.75**	**0.45**	**<.001**	0.49	0.37	.408
(CS‐WW)–(CS‐CW)	**2.65**	**0.48**	**<.001**	0.35	0.37	.648
(WS‐WW)–(CS‐CW)	**2.40**	**0.48**	**<.001**	**0.97**	**0.37**	**.025**

*Note*: The table shows estimated marginal means contrasts from generalized linear mixed models (GLMMs) that included treatment as a fixed effect, and strain ID and batch ID as random effects (see Methods for more detail). The type of model is indicated above the contrasts: Gaussian (“*gaussian*”), negative binomial with linear parametrization (“*nbinom1*”), negative binomial with quadratic parametrization (“*nbinom2*”), or binomial (“binomial”). *p*‐values are after Dunnett's correction for multiple comparisons.

Statistically significant differences are highlighted in bold.

### 3.3 Sexual fitness

The number of gonads produced was also significantly influenced by treatment (males: LRT, Gaussian GLMM, *λ* ^2^ = 56.43, *p* < .001; females: LRT, negative binomial GLMM with linear parametrization, *λ* ^2^ = 34.66, *p* < .001). Male polyps exposed to a simulated summer heatwave had reduced number of testes (Table 1; Figure 3b). The negative effects of a warm summer were accentuated if they were followed by control winter: a sudden large drop in temperature was associated with markedly reduced male fitness (Table 1; Figure 3b). In females, on the contrary, only the WS‐CW group had significantly reduced number of eggs compared with the CS‐CW group (Table 1, Figure 4b).

Surprisingly, polyps exposed to a warm winter treatment underwent a second round of gametogenesis after the first one. This second gametogenesis event happened in individuals that recovered from their first gametogenesis event, around 3–4 months after cooling (Figures 3c and 4c).

### 3.4 Asexual fitness

**FIGURE 5:**
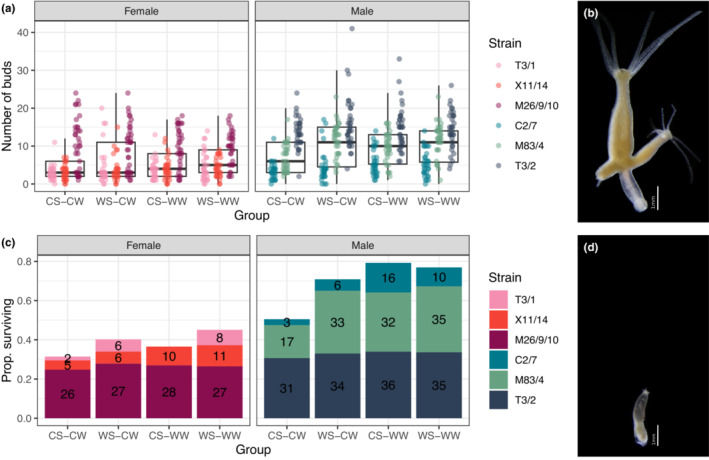
Asexual reproduction and survival in *Hydra oligactis* polyps belonging to three male and three female strains, exposed to four distinct temperature regimes: CS‐CW (18°C summer–8°C winter; control group), WS‐CW (23°C summer–8°C winter), CS‐WW (8°C summer–12°C winter) or WS‐WW (23°C summer–12°C winter). The summer treatment lasted for one week, while the winter treatment lasted 21 weeks. The graph shows the total number of buds produced during the 22 weeks of the experiment (a) and proportion of individuals surviving (c). Photographs of an asexual individual (b) and a senescent individual (d) are shown for illustration.

In males, asexual fitness was significantly affected by treatment (LRT, negative binomial GLMM with linear parametrization, *λ* ^2^ = 26.96, *p* < .001). The number of buds produced increased if polyps were exposed to a simulated summer heatwave and if they were cultured under elevated winter temperatures, or they received both treatments compared with a control summer‐control winter scenario (Table 1, Figure 5a). In females, by contrast, treatment had no effect on asexual fitness (LRT, negative binomial GLMM with quadratic parametrization, *λ* ^2^ = 6.11, *p* = .106).

### 3.5 Survival rate

Male survival rate was significantly affect by treatment (LRT, Binomial GLMM, *λ* ^2^ = 44.36, *p* < .001). Male polyps exposed to a simulated summer heatwave, elevated winter temperature, or both treatments had significantly higher survival than polyps in the control group (Table 1, Figure 5c). The highest survival rate was seen in male polyps exposed to the CS‐WW treatment, where about 80% of the polyps survived compared to ~50% in the control CS‐CW group. Female survival was only marginally significantly affected by treatment (LRT, Binomial GLMM, *λ* ^2^ = 7.36, *p* = .061), with the WS‐WW group being significantly more likely to survive compared with the control group (~45% of female polyps survived compared to 30% in the control group; Table 1, Figure 5c).

## 4 DISCUSSION

Organisms are heavily affected by warming temperatures and show short‐ or long‐term biological responses that include advancing phenologies, shifting geographical distribution ranges, and altered physiology (Huey & Kingsolver, 2019; McCarty, 2001; Thomas, 2010; Walther et al., 2002). Warming also affects biological fitness, such that organisms have altered reproduction and/or survival, with important consequences on population dynamics and ecosystem stability (Anderson, 2016; Deutsch et al., 2008; Kingsolver et al., 2013; Sinclair et al., 2016). While the fitness consequences of warming are widely considered to be negative, detailed investigations show a much more varied picture, with both negative and positive effects observed depending, for example, on the thermal tolerance and phenotypic plasticity of the species (Chamaillé‐Jammes et al., 2006; Deutsch et al., 2008; Pelini et al., 2012; Weitere et al., 2009).

Here, I studied the fitness consequences of warming across the life cycle of a facultatively sexual freshwater cnidarian *H. oligactis*, an important predator of zooplankton in freshwater ecosystems. This species reproduces asexually during summer, switches to a sexual mode of reproduction after the temperature drops during autumn and experiences a postreproductive senescence with increased mortality risk (Sebestyén et al., 2018, 2020). However, some individuals do not initiate sexual reproduction at all, or survive and continue reproducing asexually during the winter. Due to the heat intolerance of this species (Bosch et al., 1988) and the dependence of sexual reproduction on cold temperature in *H. oligactis*, I hypothesized that warming might have negative fitness effects and an overall shift from sexual to asexual reproduction. The results paint a more complex picture of fitness effects of warming in *Hydra*.

Exposure of hydra polyps to a simulated summer heatwave had immediate negative effects on the body size of hydra polyps. The reduction of body size with warming in animals is a widespread phenomenon (Gardner et al., 2011; Sheridan & Bickford, 2011) and is thought to be due to altered metabolic requirements and nutrient dependence (Audzijonyte et al., 2022; Lee et al., 2015), oxygen limitation (Verberk et al., 2021; Walczyńska et al., 2015) or temperature‐dependent size–fecundity or size–mortality relationships (Arendt, 2015; Audzijonyte et al., 2022). The size declines observed here were the result of phenotypic flexibility and occurred very quickly (observed after a week of exposure to simulated heatwave), suggesting a likely involvement of heat stress in the shrinkage of hydra polyps. Although such an immediate reduction in body size due to heat stress might not occur in most animals with a relatively fixed adult body size (e.g., insects), several other groups are able to plastically change adult size in response to environmental conditions (see, e.g., Thommen et al., 2019 for an example of body size fluctuations in response to food availability in planarians), and in these animals warming, and the associated heatwaves could have an immediate effect on body size, just like in hydra. Furthermore, the changes in body size in hydra are significant because body size at cooling is a strong predictor of subsequent performance in this species: small polyps have delayed sexual maturation, reduced fecundity, but a higher postreproductive survival (Ngo et al., 2021). In accordance, I found that polyps in the WS groups, exposed to a simulated summer heatwave required more time to produce the first gonads, had reduced sexual fitness and at least in males, they had a higher postreproductive survival rate and higher asexual fitness.

The effects of the summer heatwave, however, were modulated by the winter treatment in a complex way. In *H. oligactis*, temperatures below 12°C degrees are required for gamete differentiation to occur (Littlefield, 1991; Littlefield et al., 1991), and researchers previously used temperatures from 4 to 10°C to induce sex in this species (Boutry et al., 2022; Kaliszewicz, 2015; Tomczyk et al., 2020). Higher simulated winter temperatures advanced sexual maturation in both males and females, which is consistent with a hypothesis that higher temperatures are more permissive for cell proliferation and the differentiation of gametes and reproductive tissue (Álvarez & Nicieza, 2002). These results suggest that, while all temperatures below or equal to 12°C are able to promote sexual development in *H. oligactis*, the exact temperature can affect fine details of sexual development. The strongest effects on sexual development and fecundity were, however, observed in the WS‐CW group, where the simulated summer heatwave was followed by a sudden drop in temperature. Animals in this group needed the most time to produce the first gonads and, at least in males, they had the lowest number of reproductive organs. This suggests that sudden drops in temperature are stressful for Hydra, and this stress could contribute to sex induction in this species. While temperatures as large as this (from 23 to 8°C within one day) are unlikely to occur under natural conditions in a freshwater habitat, both heatwaves and cold spells, that is, temperature variability are predicted to be more common in the future (Meehl et al., 2007) and these are likely to have negative physiological consequences in Hydra. Remarkably, I detected an unexpected consequence of simulated winter warming: polyps cultured under 12°C underwent a second round of gonadogenesis and continued to show signs of sexual reproduction five months after cooling, while all surviving polyps cultured on 8°C were asexual. Hence, while warming might have immediate negative effects on sexual reproduction in general in this species (especially if it demonstrates in increased temperature fluctuations), these negative effects could be counterbalanced on the longer term by the positive effects of warm winters on the number of reproductive cycles. It must be emphasized, however, that the multiple rounds of gonadogenesis observed here should be dependent on the length of the cold winter period, since the second round of gametogenesis started about four months after cooling on a constant temperature. It remains to be shown whether similar patterns will be observed under more realistic temperature regimes.

Compared with sexual reproduction, the effects of elevated temperatures on asexual fitness were much more clear‐cut (although sex‐dependent). Polyps exposed to a simulated heatwave produced a higher number of asexual buds in male strains, and males exposed to elevated winter temperatures produced a higher number of asexual buds. Although the effects in females were subtle, and the male strains showed substantial variability, the overall effect was clear. The increased asexual fitness might be explained on one hand by a shift from sexual to asexual reproduction (possibly mediated by the reduced size of polyps exposed to a simulated heatwave) and also by the higher survival rate of polyps exposed to higher temperatures. Since asexual reproduction allows very quick population growth, these results suggest that increased temperatures will result in higher hydra population sizes in late winter due to climate warming. Hence, temperate freshwater bodies might experience “hydra blooms” similar to the warming‐induced jellyfish blooms observed in marine environments, given sufficient food will be available to them (Goldstein & Steiner, 2020; Holst, 2012; Purcell et al., 2007). Ultimately, this could have potential up and downstream consequences on the whole aquatic food web, such as increasing predation pressure on freshwater zooplankton. Unfortunately, little is known about hydra population dynamics under natural settings. Based on the phenology of resting egg production in *H. oligactis*, which peaks before the onset of the winter, one could assume that population size collapses during winter either due to freezing, low food availability, or increased mortality due to reduced somatic maintenance of sexually reproducing polyps (Sebestyén et al., 2018). The limited number of field observations, however, seems to contradict this assumption, since all observations point to the fact that *H. oligactis* thrives during the winter and reaches very large population densities (Bryden, 1952; Ribi et al., 1985; Welch & Loomis, 1924). The experiment presented here suggests that Hydra population sizes could become even higher in the future due to climate warming. Future studies should aim to test these predictions in a more natural setting, for example, in mesocosms simulating the complexity of an aquatic food web to gain more insight into this problem.

In parallel with the higher asexual fitness of polyps exposed to simulated warming, I also found a positive effect of summer heatwave and warmer winters on postreproductive survival. Indeed, the higher asexual fitness of polyps exposed to higher temperatures might be at least partly consequence of their increased survival rate, although this effect was observed only in males, but not in females. Hence, temperature appears in a growing list of intrinsic and extrinsic factors that affect postreproductive survival in this species (age, size, genotype; Miklós et al., 2022; Ngo et al., 2021; Sebestyén et al., 2020). Exposure to high temperature could have resulted in higher survival for at least two different reasons. First, simulated a summer heatwave caused a reduction in body size, which is known to directly influence survival rate in this species (Ngo et al., 2021), most likely through a shift of resources from reproduction to survival. However, even male polyps that were not exposed to a simulated summer heatwave experienced improved survival rate if they were cultured at 12°C simulated winter temperatures, which points at a second, independent mechanism. The higher temperature could have caused improved survival by altering the metabolic cost of tissue maintenance in these polyps (Gillooly et al., 2001). Alternatively, it could also be the consequence of a shift from reproduction to survival functions in polyps experiencing warmer winters, although no clear evidence of that was observed, since polyps in the “Warm Winter” groups did not show evidence of reduced testes number compared with the “Control Winter” groups.

To conclude, increasing temperature due to global warming is expected to affect multiple fitness components in *H. oligactis*. While sexual fitness might not be reduced, winter survival and asexual reproduction is expected to increase, potentially resulting in higher Hydra population size and increased predation on freshwater zooplankton and fish larvae. Further studies should explore the effect of temperature on postreproductive survival in more detail, potentially in combination with other abiotic factors that could exacerbate the effects of warming (e.g., acidification and salinization; Zalizniak et al., 2009).

## AUTHOR CONTRIBUTIONS

Jácint Tökölyi contributed to conceptualization (lead); data curation (lead); formal analysis (lead); funding acquisition (lead); methodology (lead); project administration (lead); visualization (lead); and writing (lead).

## Data Availability

Raw data are available on Figshare (10.6084/m9.figshare.21750188.v1). R code to analyze the data is available on Figshare (10.6084/m9.figshare.21750170.v1).
